# Antibacterial and Biocompatible Penicillin–Streptomycin Loaded Bacterial Cellulose (BC) Hydrogels for Wound Healing

**DOI:** 10.3390/gels11110851

**Published:** 2025-10-24

**Authors:** Sanosh Kunjalukkal Padmanabhan, Maria Elena Giordano, Stefania Villani, Gayatri Udayan, Mariangela Stoppa, Pietro Alifano, Christian Demitri, Maria Giulia Lionetto, Antonio Licciulli

**Affiliations:** 1 Department of Experimental Medicine, University of Salento, 73100 Lecce, Italy; pietro.alifano@unisalento.it (P.A.);; 2 Department of Biological and Environmental Sciences and Technologies (DiSTeBA), University of Salento, 73100 Lecce, Italy; elena.giordano@unisalento.it (M.E.G.); gayatri.udayan@unisalento.it (G.U.); giulia.lionetto@unisalento.it (M.G.L.); 3 Department of Engineering for Innovation, University of Salento, 73100 Lecce, Italy; stefania.villani@unisalento.it (S.V.);

**Keywords:** bacterial cellulose, antibiotic, hydrogel, wound healing, antibacterial

## Abstract

Bacterial cellulose (BC) hydrogel is a promising skin wound healing biomaterial due to its unique properties, including a moist environment that facilitates tissue healing. To enhance its antimicrobial efficacy, BC dressings were loaded with penicillin and streptomycin. FT-IR analysis confirmed successful drug binding, while SEM revealed a nanofibrous and porous hydrogel structure. In vitro studies using 3T3 mouse fibroblasts demonstrated biocompatibility, and scratch wound assays achieved complete closure across all tested concentrations. Antibacterial activity, assessed via agar diffusion against *Pseudomonas aeruginosa* and *Staphylococcus aureus*, showed a concentration-dependent increase in inhibition zones, highlighting the potential of BC-Pen/Strep hydrogels as effective antimicrobial wound dressings.

## 1. Introduction

Human skin is a multifunctional organ that serves as both a physical and functional barrier between the internal body and the external environment [1]. It provides the body’s primary defense against external pathogens and also prevents body dehydration [2]. When it is injured by physical, thermal, chemical, or mechanical trauma, the protective barrier is compromised, increasing susceptibility to infection and fluid loss [3,4]. Effective wound care is essential to prevent contamination and facilitate the rapid healing and regeneration of the damaged skin. Chronic wounds often become colonized by a diverse and high load of bacteria, which is a major factor in delaying the healing process [5]. The most common pathogens related to chronic wound infections are *Staphylococcus aureus* and *Pseudomonas aeruginosa*, found in surgical site infections, diabetic foot ulcers, and burns [6]. *S. aureus* is a Gram-positive opportunistic pathogen, and its treatment is often complicated by the emergence of antibiotic-resistant strains, like the MRSA (methicillin-resistant *S. aureus*) [7]. *P. aeruginosa* is a Gram-negative bacterium that plays a crucial role in the clinical course of patients affected by chronic skin lesions, mainly due to the tendency to develop biofilms, contributing to adaptive resistance [8,9].

Novel therapeutic approaches, such as the use of biopolymers, are needed to effectively treat these infections. Biopolymer-based hydrogels are promising wound dressing materials owing to their capacity to maintain moisture, absorb exudate, offer softness, respond to stimuli, and enable controlled drug delivery [10].

Bacterial cellulose (BC) is a biopolymer with diverse applicability in the food and biomedical industries, produced through the fermentation of the Gram-negative bacterium *Acetobacter xylinum*, which generates high-aspect-ratio nanofibers with a three-dimensional (3D) porous network. Unlike other materials, BC does not require complex extraction methods or harsh chemical treatments while maintaining exceptional purity. Its unique 3D web-like nanofibrous structure gives it outstanding mechanical properties and moldability, particularly in its wet form, making it easy to shape as needed. With its hydrophilic nature (comprising 99% water), flexibility, nontoxicity, excellent biocompatibility, and wide availability, BC has gained attention as an advanced wound dressing due to its natural ability to accelerate wound healing [11]. Cheng et al. reported on motion-responsive injectable hydrogels designed for dynamic tissue environments, highlighting adaptability as a key feature relevant to flexible BC-based dressings [12]. Zhang et al. developed bioinspired Kevlar/hydrogel composites combining mechanical toughness and sensing capabilities, offering insights for enhancing the durability of BC hydrogels [13]. Similarly, Wang et al. reported MXene-based responsive hydrogels that promote accelerated healing, providing inspiration for developing advanced drug-releasing BC systems [14].

One of the essential properties of wound dressing materials is their ability to prevent microbial infections, thereby accelerating wound healing and further skin remodeling [15]. However, BC lacks antimicrobial activity, limiting its use in infectious wound care. On the other hand, its porous nanofibrillar structure allows for impregnation with antimicrobial agents, overcoming this limitation for use in infectious wound dressings [11]. Various organic substances (e.g., antibiotics, antimicrobial peptides) and inorganic nanoparticles have been incorporated into the BC network to impart antibacterial properties.

The increasing prevalence of antibiotic resistance poses a major global health concern, while the pace of new antibiotic discovery has markedly declined in recent decades. Consequently, the research has turned its attention to the rational development of drug combination strategies to counteract antibiotic resistance [16]. The incorporation of penicillin and streptomycin (Pen/Strep) in hydrogel is a relevant opportunity to enhance the antibacterial and antibiofilm action of biomaterials [17], promoting a co-administration to help reduce the risk of the emergence of antibiotic-resistant strains [16]. For topical application to wounds, a solution of streptomycin should be used in conjunction with penicillin because it targets and kills Gram-negative bacilli, against which penicillin is ineffective. Additionally, some of these bacteria produce penicillinase, an enzyme that breaks down penicillin, further reducing its effectiveness [18].

Thus, our study aimed to incorporate different amounts of Pen/Strep, antibiotics commonly used in clinical practice, into BC hydrogel membranes. This would enable a new route of administration for treating wounds infected by Gram-positive and Gram-negative strains, positioning Pen/Strep as a nanobiotechnological product for topical use. The developed hydrogels were characterized by their in vitro antibacterial activity against *S. aureus* and *P. aeruginosa* strains, as well as their biocompatibility and ability to promote wound closure in vitro using murine 3T3 fibroblast cells.

## 2. Results

### 2.1. Physicochemical Analyses

**Figure 1:**
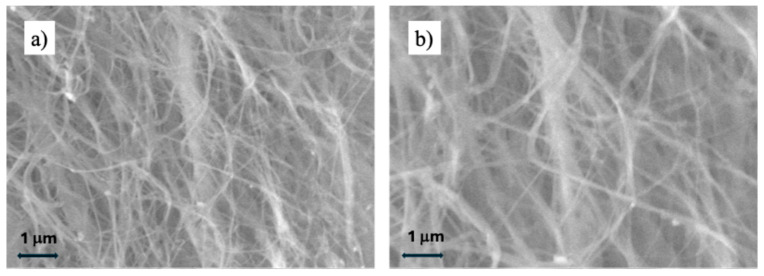
SEM images of BC, showing the fiber morphology (**a**) on the surface and (**b**) in cross-section.

SEM analysis, performed on both cross-sections and surfaces, as shown in Figure 1, revealed that BC possesses a homogeneous three-dimensional architecture as a biological nonwoven fabric made of a nanofibrillar cellulose network, in agreement with previous reports [19]. We also observed a higher fiber density in cross-section (Figure 1b) compared to surface (Figure 1a), which might be due to the compressive nature of freeze-dried hydrogel during cross-sectional slicing. The nanofibrillar network can be modeled as a material with open interconnected porosity, a feature that explains BC’s high water uptake, exudate absorption, and capacity to adsorb and release bioactive agents. Unlike cellulose aerogels, BC displays a distinctive fibrillar organization despite similarly high porosity [20].

**Figure 2:**
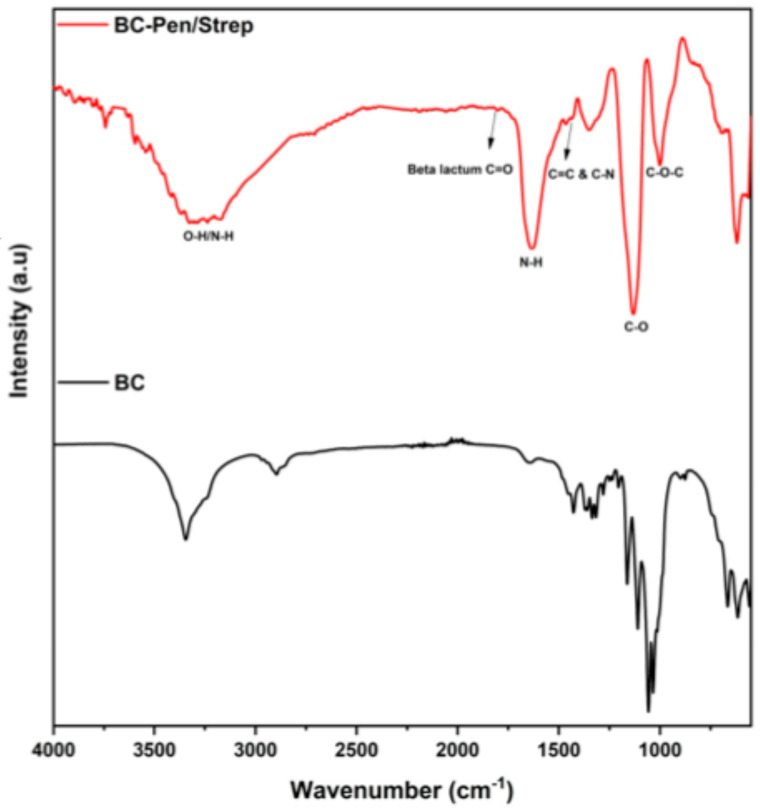
FTIR spectra of BC control and BC-Pen/Strep.

Figure 2 shows the FT-IR spectra of BC control and BC-Pen/Strep 1. FT-IR analysis of the BC control showed its characteristic chemical bonds: a stretching vibration of OH groups at 3346 cm^−1^, and a stretching vibration of the CH_2_ and CH groups at 2896 cm^−1^. The band at 1647 cm^−1^ corresponds to the H–O–H bending vibration of crystallization water, while the absorption at 1280 cm^−1^ can be attributed to the O–H deformation vibrations [19,21]. The band at 1205 cm^−1^ can be assigned to C–H deformation vibrations, whereas the absorption observed at 1160 cm^−1^ corresponds to the asymmetric stretching vibrations of C–C bonds [22]. Related to the literature, the penicillin and streptomycin spectra showed several specific absorption bands. Pen/strep-loaded BC membrane shows characteristic absorption bands of penicillin related to its β-lactam ring, thiazolidine ring, and amide/acid functional groups at 1770–1780 cm^−1^, 1450–1600 cm^−1^, 1715–1730 cm^−1^, and 1660–1680 cm^−1^, respectively [23]. Streptomycin characteristic absorption bands around 3400 cm^−1^ (O–H, N–H) combined with intense C–O–C and C–O stretches in the 1150–1000 cm^−1^ region, which reflect the aminoglycoside structure, are evident in the BC-Pen/Strep spectra [24]. FT-IR analysis provides evidence that penicillin and streptomycin molecules are absorbed onto the porous structure of BC. This study primarily focused on assessing the biological performance and compatibility of the dressings, while mechanical and physical characterization will be undertaken in future studies.

### 2.2. Cytotoxicity Assessment

**Figure 3:**
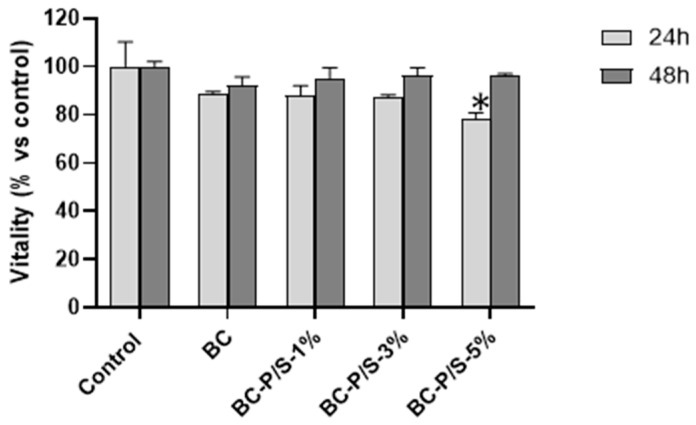
Viability of 3T3 cells after 24 h and 48 h exposure to undiluted eluates from Pen/Strep-loaded and non-loaded BC hydrogels. Data are expressed as mean ± SD. * *p* < 0.05 vs. control.

The in vitro biocompatibility of BC and Pen/Strep-loaded BC hydrogels was carried out using 3T3 mouse fibroblasts. Indirect cytotoxicity was assessed to evaluate whether substances leaching from the materials could adversely affect cellular viability. As illustrated in Figure 3, the metabolic activity of cells exposed to the hydrogel eluates for 24 h consistently remained above 80% relative to untreated control cells; in the case of 48 h exposure, the percentage increased to 90%, indicating the good health status of the cells. No significant effects were observed between control and treated cells, except for the BC-P/S-5% conditions. Furthermore, no significant differences were detected between the unloaded and Pen/Strep-loaded hydrogels across all tested concentrations. Indirect tests are particularly useful for detecting any residual chemicals, bacterial byproducts, or degradation products that might leach from BC after processing [25]. Therefore, the obtained results allowed us to exclude the presence of any residual leachable compounds in either the BC hydrogel or the Pen/Strep-loaded BC hydrogels that could cause cytotoxic effects on the cells. Moreover, this result is in agreement with literature data showing that BC typically does not release cytotoxic substances under indirect testing [26].

**Figure 4:**
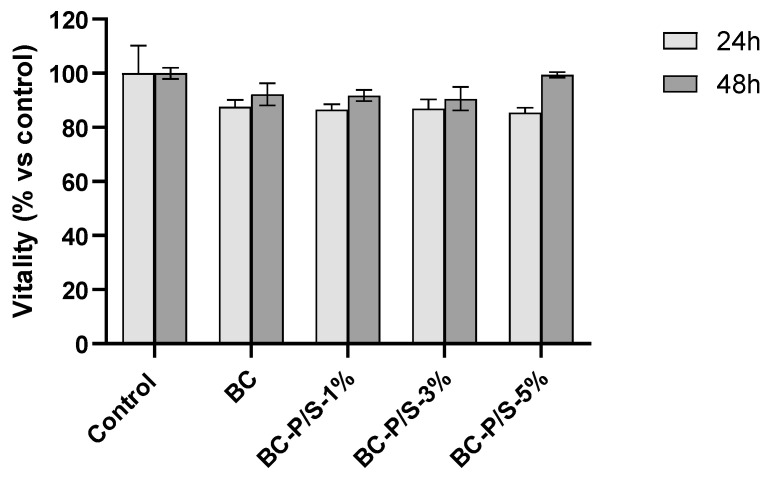
Viability of 3T3 cells after 24 h and 48 h direct contact with Pen/Strep-loaded and non-loaded BC hydrogels. Data are expressed as mean ± SD.

Direct cytotoxicity of the materials was assessed in accordance with ISO 10993-5, the standard for biological evaluation of medical devices. Cell viability was measured following 24 and 48 h of direct exposure to the samples (Figure 4). Also in this case, cell viability values of at least 85% and 90% were registered for the hydrogels after 24 h and 48 h of exposure, respectively, in comparison with the positive control. No statistically significant differences were observed between the experimental conditions and their respective controls for either the 24 h or 48 h exposure times. Similarly, no significant difference was found between the BC hydrogel and the Pen/Strep-loaded BC hydrogels at any concentration tested. This observation aligns with findings from other studies showing that both unmodified and chemically modified BC maintain high cell viability and do not induce cytotoxicity, even after several days of direct contact with mammalian cells (e.g., fibroblasts, keratinocytes, smooth muscle cells, and HEK293) [27,28,29].

**Figure 5:**
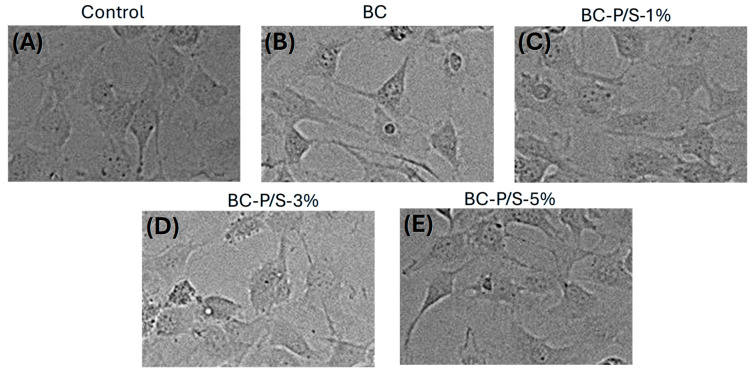
(**A**–**E**): Representative images of cells exposed for 48 h to undiluted eluates from Pen/Strep-loaded and non-loaded BC hydrogels; the cells were visualized under bright field microscopy using the multimode reader Cytation 5 (40× objective used for visualization).

Cell morphology was also evaluated through optical microscopy, as reported in Figure 5, which shows the 3T3 murine fibroblasts after 48 h exposure to undiluted eluates from BC hydrogel and the Pen/Strep-loaded BC hydrogels. It is well established that changes in the size or structure of 3T3 fibroblasts—particularly disruptions in the actin cytoskeleton and overall cell architecture—can be indicative of cytotoxic effects [30,31]. Such morphological alterations are typically associated with additional signs of cellular stress, including reduced viability [32]. In this study, the cells maintained their typical shape in all experimental conditions tested, and no visible changes in cell shape or size were observed compared to control cells, supporting the favorable results obtained from the MTT assay. Overall, MTT and morphological results point to the biocompatibility of the material tested at the applied concentrations in line with the ISO 10993-5 threshold for biocompatibility.

### 2.3. Wound Healing and Cell Migration

**Figure 6:**
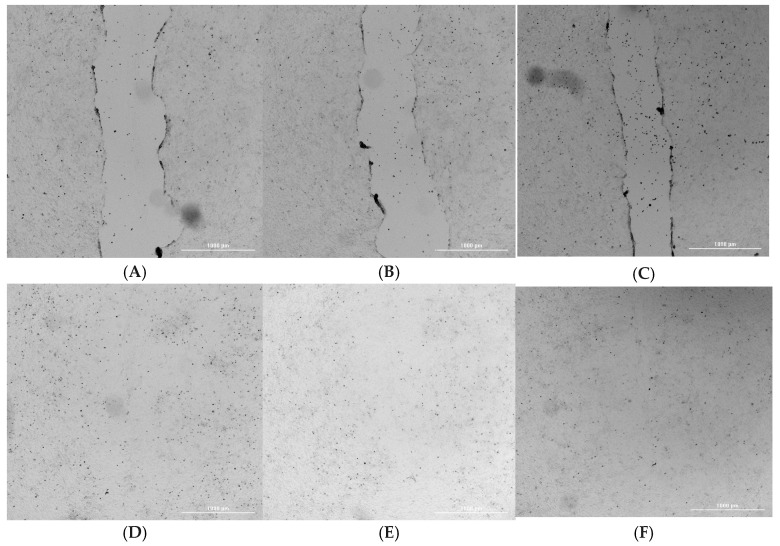
Representative bright-field images of the scratch test on NIH 3T3 cells acquired by the imaging multimode reader Cytation 5 Biotek (obj 4×). (**A**) C T0. (**B**) BC T 0. (**C**) BC-P/S-1% T 0. (**D**) C T 24 h. (**E**) BC T 24 h. (**F**) BC-P/S-1% T 24 h.

Fibroblasts play a critical role in tissue regeneration and wound repair due to their involvement in processes such as cell migration [33]. To investigate whether exposure to BC affected fibroblasts’ mobility or migratory behavior, a scratch wound assay was conducted. The scratch assay primarily evaluates cell migration and proliferation, reflecting the ability of cells to move and repopulate the wounded area [34,35,36]. Cells were incubated for 24 h with either non-loaded or P/S1%-loaded hydrogel before assessing their migration capacity. As shown in representative Figure 6A–F the exposure of the cells to the BC hydrogel did not affect cell migration or subsequent wound closure. Quantification analysis indicated a complete (100%) wound closure in all the conditions tested. The quantification of the effect corresponded to 100% wound closure percentage in all the conditions tested. This result indicates that the Pen/Strep-loaded hydrogels maintain a microenvironment supportive of fibroblast migration and proliferation, enabling complete wound closure in vitro. This is in agreement with previous studies demonstrating that BC-based dressings support fibroblast motility and enhance wound closure in vitro and in vivo, making them promising materials for advanced wound care [37,38,39].

Taken together, data obtained by either indirect and direct assay or wound healing assay demonstrate that Pen/Strep-loaded BC hydrogels are cytocompatible and do not interfere with fibroblast viability, morphology, or migration, fulfilling key prerequisites for wound dressing applications.

### 2.4. Antimicrobial Properties of Pen/Strep and Pen/Strep-Loaded BC Hydrogels

**Figure 7:**
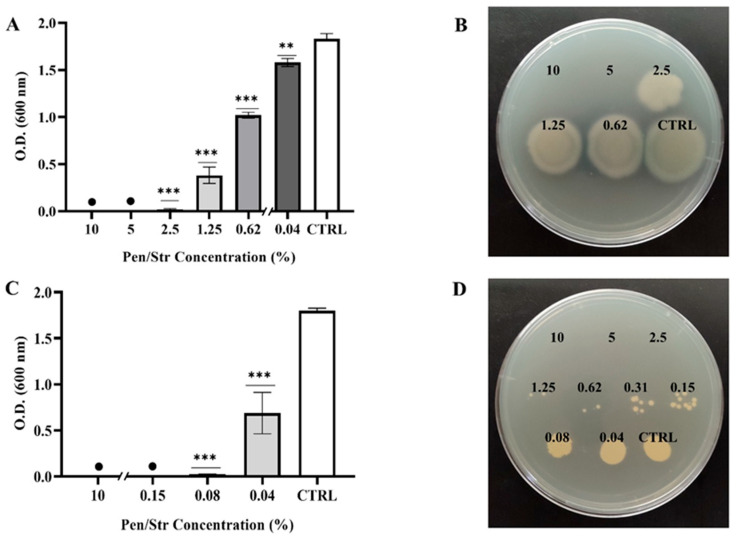
Determination of the Minimal Inhibitory Concentration (MIC) (**A**,**C**) and the Minimal Bactericidal Concentration (MBC) (**B**,**D**) for Pen/Strep solution ranging 0.04–10% against *P. aeruginosa* (**A**,**B**) and *S. aureus *(**C**,**D**). The symbol “●” indicates that no growth was observed. ** *p* < 0.01, *** *p* < 0.001 compared to the control (CTRL).

*P. aeruginosa* (Gram-negative) and *S. aureus* (Gram-positive) were chosen as two of the most common pathogens of skin lesions to assess the bacteriostatic and bactericidal properties of Pen/Strep. The results of the visual evaluation are reported in Table 1, indicating the presence of appreciable bacterial growth with “+”, and its absence with “−”. This assessment was confirmed quantitatively by determining the O.D. by spectrophotometric measurements at 600 nm. From this analysis, it was possible to establish that the MIC values for Pen/Strep are 2.5% for *P. aeruginosa* GG7 and 0.07% for *S. aureus* SA01 (Figure 7A,C). Further experiments revealed that the MBC values are 5% and 2.5% for *P. aeruginosa* and *S. aureus*, respectively, as shown in Figure 7B,D, where no visible bacterial colonies were observed at the MBC and higher concentrations.

**Figure 8:**
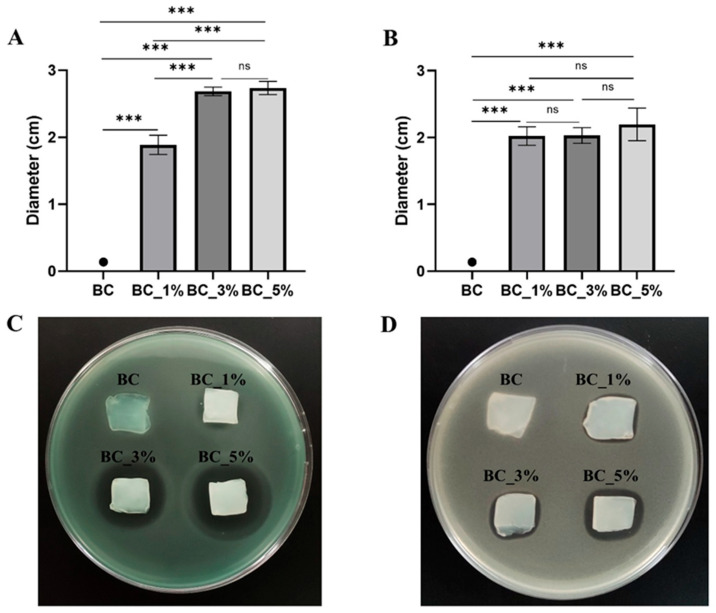
Evaluation of the antimicrobial properties of pristine and Pen/Strep-loaded BC hydrogels with 1%, 3%, and 5% Pen/Strep solution (BC-P/S-1%, BC-P/S-3%, and BC-P/S-5%, respectively) by determining the diameter of the inhibition zone for *P. aeruginosa* (**A**,**C**) and *S. aureus* (**B**,**D**). The symbol “●” indicates that no growth was observed. “ns” non significant; *** *p* < 0.001.

Three different concentrations of Pen/Strep bracketing MIC values were used to load BC hydrogels (BC-P/S-1%, BC-P/S-3%, BC-P/S-5%) and confer antibacterial properties, and the resulting efficacy was assessed by an agar diffusion test (Figure 8). Pure BC hydrogels were used as a control (BC). No halo was observed against both *P. aeruginosa* and *S. aureus*, confirming the lack of antibacterial action of the unmodified BC hydrogels, as previously reported in the literature [40]. For Pen/Strep-loaded BC hydrogels, the appearance of an inhibition halo was observed for both bacterial species, with an increase in the halo diameter as the concentration of Pen/Strep increased. For *P. aeruginosa*, the halo diameter increased from ~1.8 cm to ~2.7 cm as the concentration was raised from 1% to 5%. In contrast, for *S. aureus*, the halo diameter remained constant at around 2 cm, regardless of concentration.

The promising antibacterial performance of BC-based hydrogels functionalized through the co-loading of Pen/Strep suggests their potential as smart materials for the treatment of skin wounds complicated by microbial infections. To further enhance both the antimicrobial efficacy while preserving the biocompatibility of functionalized BC hydrogels on damaged skin, future studies will explore co-loading with Reactive Oxygen Species (ROS)-generating agents, such as plasma-activated water [41,42,43]. 

Another promising approach to further exploit the performance of functionalized BC-based hydrogels is to engineer them into smart systems capable of responding to stimuli from the surrounding microenvironment. By conferring spatial and temporal responsiveness, these systems can achieve a higher level of adaptivity compared to conventional hydrogels [44,45,46,47]. In the context of wound healing applications, in particular, BC hydrogels lend themselves well to chemical modifications that enable pH-responsive swelling [46,48] or aptamer-mediated response by enabling real-time sensing and triggering a responsive release of antimicrobial agents [49,50].

## 3. Conclusions

This study aimed to explore the potential of bacterial cellulose loaded with varying amounts of penicillin and streptomycin as an antimicrobial wound dressing. Bacterial cellulose hydrogels are naturally bacteriostatic due to their non-woven fibrous microstructure, which is not passable by bacteria. In vitro evaluation of Pen/Strep-loaded BC hydrogels using 3T3 mouse fibroblasts demonstrated their biocompatibility. A scratch wound assay was performed, showing cell migration and 100% wound closure at all the concentrations tested.

The antimicrobial activity was induced by loading two antibiotics in the bacterial cellulose support by adsorption. The growth rate of *P. aeruginosa* and *S. aureus* was lower in the presence of the bacterial cellulose loaded with antibiotics (penicillin and streptomycin) due to the release of the antimicrobial agents. The agar diffusion test confirmed the lack of antibacterial action in pure BC hydrogels. In contrast, for Pen/Strep-loaded BC hydrogels, an inhibition halo was observed for both bacterial species, with increasing diameter as the concentration of Pen/Strep increased. Based on these data, it can be concluded that penicillin- and streptomycin-loaded BC represents a highly promising antimicrobial wound dressing, exhibiting long-term stability and potential for large-scale production and clinical adaptation.

## 4. Materials and Methods

### 4.1. Materials

Black tea bags, sucrose, and vinegar used were all commercial products, while ”SCOBY” (symbiotic culture of bacteria and yeast) was a proprietary product of Biofaber srl (Brindisi, Italy). Sodium hydroxide (NaOH) was purchased from Sigma-Aldrich (Steinheim, Sweden). Penicillin–streptomycin (Pen/Strep) solution from Sigma Aldrich (Saint Louis, MO, USA), having 6 mg of penicillin and 10 mg of streptomycin per mL, was used as an antibacterial agent.

### 4.2. Methods

#### 4.2.1. Bacterial Cellulose Production

Bacterial cellulose pellicles were obtained from kombucha strains by the fermentation process of the sweet black tea with *Gluconacetobacter xilinus* strains. The tea fungus, composed of an upper cellulosic pellicle and a lower liquid broth, was activated every 2 weeks according to a procedure inspired by the work of Chen and Liu but with some modifications [40]. The culture medium was prepared by adding sucrose and tea bags to boiling water (1 L). After removing the tea bags, the pH value of the broth was adjusted to ∼3.0 by adding acetic acid. Finally, the cellulosic pellicle pieces and liquid broth of the tea fungus were added to the cooled tea broth. The fermentation and BC growth were carried out at room temperature (28 °C) for 21 days in static conditions. Cellulose hydrogels formed on the surface of the broth. The pellicles were washed with distilled water and soaked in 0.5 M NaOH solution at 80 °C for 120 min under mild shaking conditions to remove any attached cells and impurities. The BC was rinsed in distilled water to remove NaOH and stored.

#### 4.2.2. Pen/Strep Loaded Bacterial Cellulose Production

BC for Pen/Strep loading was made by removing 90% of its water content and rehydrating BC hydrogels with antibiotic solutions. The partially dehydrated BC films were immersed in solutions of Pen/Strep with three different concentrations (1%, 3%, and 5% wt%) until their weight doubled. These partially hydrated and Pen/Strep-loaded BC hydrogels were placed in a cool place and used for further characterization. The samples were named as BC-P/S-1%, BC-P/S-3%, and BC-P/S-5% according to the loading concentration of antibiotics.

### 4.3. Characterization

The antibiotic-loaded BC hydrogels were characterized by advanced physicochemical analyses, which were further corroborated by systematic evaluations of their biocompatibility and antimicrobial efficacy.

#### 4.3.1. Physicochemical Analyses

Freeze-dried BC hydrogels were used for both Fourier Transform Infrared Spectroscopy (FTIR) and Scanning Electron Microscope (SEM) analysis.

The morphology of the BC was analyzed with a Zeiss (Sigma VP, Carl Zeiss, Jena, Germany) Field-Emission Scanning Electron Microscope (FE-SEM). FTIR was carried out on a Nicolet 6700 spectrometer (Thermo Fisher Scientific Inc., Waltham, MA, USA) in a diffuse reflectance setup, accumulating 36 scans over a 4000–400 cm^−1^ wavenumber range.

#### 4.3.2. In Vitro Biocompatibility Assessment by MTT Assay

The biocompatibility of the Pen/Strep BC hydrogels was tested in vitro on murine fibroblasts (NIH/3T3 cell line (ATCC^®^ CRL-1658™) LGC Promochem, Wesel, Germany) using direct and indirect methods to evaluate potential cytotoxic effects. Cell viability was assessed by MTT assay on NIH/3T3 cells, which are widely used as an experimental cellular model for in vitro biocompatibility assessments of new materials [51]. They are employed across various studies to evaluate cell proliferation, morphology, and cytotoxicity, making them a versatile cellular tool [52,53,54]. Cells were cultured in Dulbecco’s Modified Eagle Medium (D-MEM) with 10% fetal bovine serum, 2 mM L-glutamine, and 100 µg/mL Pen/Strep under 5% CO_2_ at 37 °C in a humidified incubator.

#### 4.3.3. Indirect Assay

Indirect cytotoxicity testing involves exposing cells to the medium that has been conditioned by contact with the hydrogels. This method is widely used to assess whether any leachable substances from BC and functionalized BC could be cytotoxic to cells [26].

To assess indirect cytotoxicity, 1 cm^2^ samples of BC and Pen/Strep-loaded BC hydrogels (in triplicate) were sterilized by autoclaving and placed in a 24-well plate containing 1 mL of growth medium, then incubated for 24 h at 37 °C in a 5% CO_2_ atmosphere. On the same day, cells were seeded at a density of 2 × 10^4^ cells/mL in a tissue culture-treated 96-well plate. After 24 h, the growth medium was replaced with the hydrogel-conditioned medium. Non-conditioned medium served as the positive control, while pure dimethyl sulfoxide (DMSO) was used as the negative control. Cells were subsequently incubated for 24 h and 48 h at 37 °C under 5% CO_2_.

At each experimental time point, cell viability was assessed through the MTT assay (3-(4,5-dimethylthiazol-2-yl)-2,5-diphenyltetrazolium bromide), which evaluates cellular metabolic activity. This method relies on the mitochondrial oxidoreductase enzymes present in viable cells, which convert the yellow MTT reagent into insoluble purple formazan crystals [18]. These were subsequently dissolved in DMSO, and the absorbance of the resulting solution was measured spectrophotometrically at 570 nm using a Cytation 5 plate reader (BioTek Instruments, Winooski, VT, USA). Cell viability was then calculated as follows:

% Relative viability of cells = (treated cell OD/control cell OD) × 100(1)

Representative images of the cells at the different experimental exposure conditions were acquired using a multimode reader, Cytation 5, with bright field microscopy (40× objective for visualization).

#### 4.3.4. Direct Assay

Direct-contact cytotoxicity was evaluated following the ISO 10993-5 guidelines [55] for the biological assessment of medical devices. Direct assay is a standard, reliable method for assessing biocompatibility [27]. Both unloaded and Pen/Strep-loaded BC hydrogels were tested in triplicate. Individual 1 cm^2^ samples were sterilized by autoclaving and pre-incubated with 1 mL of growth medium for 24 h at 37 °C in a humidified atmosphere containing 5% CO_2_. Concurrently, 5 × 10^4^ cells per well were seeded in a 24-well plate and cultured with 1 mL of growth medium under identical conditions. After 24 h, the medium was aspirated, and the hydrogel samples were carefully placed in direct contact with the cell monolayer. Subsequently, 1 mL of fresh growth medium was added to each well. Wells containing only growth medium represented the positive control, whereas wells containing pure DMSO represented the negative control. The plates were then incubated at 37 °C in a 5% CO_2_ atmosphere, and cell viability was determined after 24 h and 48 h using the MTT assay.

#### 4.3.5. Wound Healing and Cell Migration Assay

To evaluate the spreading and migratory behavior of 3T3 fibroblasts following exposure to Pen/Strep-loaded hydrogels, a scratch wound assay was conducted. NIH 3T3 cells were seeded in six-well plates at a density of 5 × 10^5^ cells per well in DMEM and cultured at 37 °C in a 5% CO_2_ atmosphere until reaching approximately 80% confluence. A sterile 200 µL pipette tip was then used to generate a linear scratch across the cell monolayer, mimicking a wound. The culture medium was replaced to eliminate any detached cells or debris. Then, the cells were exposed to the conditioned medium (see above) for 24 h. Images of the scratch area were captured at the same spot in each well, both before treatment and after the 24 h incubation, using a Cytation 5 multimode reader.

Wound closure was quantified by analyzing the images using ImageJ v.1.53 (National Institutes of Health, Bethesda, MD, USA) software equipped with the Wound Healing Size Tool plugin [35]. This tool automatically detects the wound boundaries, compensates for any angular deviation of the scratch, and measures several parameters, including total wound area, wound area fraction, mean wound width, and variation in wound width.

The percentage of wound closure was then calculated based on the method described by Grada et al. [34], using the following formula:

(2) $$\mathrm{Wound}\;\mathrm{closure}\;\%=\frac{A_{t=0}-A_{\Delta t}}{A_{t=0}}\times 100$$

#### 4.3.6. Study of the Antimicrobial Properties Against *Pseudomonas aeruginosa* and *Staphylococcus aureus*

*Pseudomonas aeruginosa* GG7 [56,57] and *Staphylococcus aureus* SA01 [6] were employed to assess the antimicrobial activity of the materials analyzed in this work. Both bacterial strains were cultured in Luria–Bertani (LB) broth, which contains 1% Sodium Chloride (NaCl), 1% Tryptone, 0.5% Yeast Extracts, and 1.5% Agar for solid medium. The culture medium was sterilized by autoclaving at 121 °C for 20 min before use. For each strain, a pre-inoculum was prepared by suspending an isolated colony from an 18 to 24 h LB agar plate in fresh broth and incubating overnight (ON) at 37 °C with stirring at 120 rpm. The day after, the bacterial suspension was adjusted to an Optical Density (O.D.) of 0.3–0.4 at 600 nm and used as the inoculum for further experiments.

#### 4.3.7. Determination of Minimal Inhibitory Concentration (MIC) and Minimal Bactericidal Concentration (MBC)

To evaluate the antimicrobial effectiveness of the Pen/Strep solution, the standard broth dilution method (CLSI M07-A8) was adopted. The Minimal Inhibitory Concentration (MIC) refers to the lowest concentration of an antimicrobial agent that inhibits the visible growth of a microorganism in a broth culture (Methods for Dilution Antimicrobial Susceptibility Tests for Bacteria That Grow Aerobically, 12th edition, Clinical Laboratory Standards Institute, Wayne, PA, USA, 2024). Serial two-fold dilutions of Pen/Strep solution, ranging from 0.04% to 10%, were prepared to determine the MIC. The samples were incubated ON at 37 °C, with the control (CTRL) consisting of inoculated broth without the antimicrobial agents. Following incubation, the turbidity of each tube was assessed visually and measured spectrophotometrically at 600 nm.

To determine the Minimal Bacterial Concentration (MBC), a 10 µL aliquot from each tube was sub-cultured on LB agar and incubated ON at 37 °C [58]. The following day, the MBC was determined as the lowest concentration at which no visible bacterial colonies appeared.

The experiments were conducted in triplicate.

#### 4.3.8. Agar Diffusion Method for Antimicrobial Activity Characterization

The antibacterial properties of pure and Pen/Strep-loaded BC hydrogels were evaluated using the agar diffusion method [59]. Following this method, each sample was cut into 2 × 2 cm square pieces, UV-sterilized for 10 min, and placed on an LB agar plate inoculated with a 0.5 McFarland-equivalent bacterial suspension, as reported by the guidelines outlined in the CLSI M100—Performance Standards for Antimicrobial Susceptibility Testing (M02—disk diffusion) [59]. Plates were incubated ON at 37 °C, after which the diameter of the inhibition zone was measured using ImageJ v.1.53 (National Institutes of Health, Bethesda, MD, USA) software. The experiments were conducted in triplicate.

### 4.4. Statistical Analysis

Data are presented as the mean ± standard deviation for the indicated number of experiments. The statistical analysis was conducted by using the one-way ANOVA technique, followed by multiple comparisons to the control (CTRL). The differences were statistically significant when * *p* < 0.05, and the significance level was reported when present.

## Figures and Tables

**Table 1:** Estimation of the bacterial growth in MIC experiments for Pen/Strep by visually estimating the suspension turbidity (+ higher, − lower).

Pen/Strep Concentration (%)										
	10	5	2.5	1.25	0.62	0.31	0.25	0.08	0.04	CTRL
***P. aeruginosa***	−	−	−	+/−	+	+	+	+	+	+
***S. aureus***	−	−	−	−	−	−	−	−	+	+

## Data Availability

The original contributions presented in this study are included in the article. Further inquiries can be directed to the corresponding author.
